# A Low-Noise High-Resolution Temperature Measurement Technique Based on Inductive Voltage Divider and Alternating-Current Bridge

**DOI:** 10.3390/s25092777

**Published:** 2025-04-28

**Authors:** Shanghua Gao, Xiaoyi Zhu, Xiaofeng Zhang, Bing Xue, Jilou Xi, Jiang Li, Bing Zhang, Xiaolei Wang, Yuru Wang, Haoyue Zhang, Xu Wu

**Affiliations:** 1 Institute of Earthquake Forecasting, China Earthquake Administration, Beijing 100036, China; gao966@ief.ac.cn (S.G.); xueb@ief.ac.cn (B.X.); xjl@ief.ac.cn (J.X.); lijiang@ief.ac.cn (J.L.); zhangbing@ief.ac.cn (B.Z.); wxl@ief.ac.cn (X.W.); wyr425@126.com (Y.W.); zhanghaoyuedq@163.com (H.Z.); 2 Key Laboratory of Earthquake Forecasting and Risk Assessment, Ministry of Emergency Management, Beijing 100036, China; 3 Innovation Academy for Microsatellites, Chinese Academy of Sciences, Shanghai 201203, China; wuxu@microsate.com

**Keywords:** temperature measurement, low noise, alternating-current (AC) bridge, inductive voltage divider, discrete Fourier transform

## Abstract

In the field of space gravitational wave detection, high-precision temperature measurement with a resolution at the micro-Kelvin level in the milli-Hertz frequency range is required to mitigate the interference caused by temperature fluctuations around the core components. This is a very challenging task due to resistance thermal noise and the inherent 1/f noise of electronic components. To overcome this problem, this paper proposes a low-noise, high-resolution temperature measurement method based on an inductive voltage divider and an alternating-current (AC) bridge. The proposed method has the following three characteristics: (1) it employs an AC excitation signal to drive the temperature measuring bridge to overcome the influence of 1/f noise in electronic components; (2) it uses as few resistance components as possible in the AC bridge and signal detection circuit to reduce the impact of resistance thermal noise on the measurement results; (3) it adopts a frequency-domain data processing algorithm based on discrete Fourier transform to improve the accuracy of the temperature measuring result. Using this method, a circuit board is designed and tested. The results show that the noise floor level of the designed temperature measurement circuit is below $7\times 10^{-6}\;K/\sqrt{\mathrm{Hz}}$ in a frequency range of 0.005~1 Hz. This demonstrates that our proposed method is able to detect extremely weak temperature change signals and meets the temperature measurement requirements of space gravitational wave detection.

## 1. Introduction

As human exploration of outer space deepens, various observational and experimental instruments have been placed in space to conduct physical experiments, astronomical observations, and research into the origin and history of the universe. As observational targets extend toward the macroscopic and microscopic extremes, the need for various indicators on satellites grows, and precise temperature measurements and control are particularly critical [1,2].

China’s “Taiji Project”, which aims at gravitational wave detection, requires measurement of the characteristic strain reaching a minimum on the order of 10^−21^ at an arm length of 3 million kilometers. Temperature fluctuations around the core components, such as the inertial sensor electrode cage and the optical platform of the optical measurement system in inter-satellite laser interferometry technology, can significantly interfere with gravitational wave detection. These interferences include forces and stiffness changes impacting the inspection quality of the inertial sensor and influencing the optical path length of the interferometer in optical measurement systems. To mitigate the above interferences caused by temperature fluctuations, the core components are required to maintain extremely high temperature stability, that is, ≤10^−5^ K/Hz^1/2^ @1 mHz~0.1 Hz [3,4]. The Taiji-1 satellite, a verification satellite for gravitational wave detection technology, uses a three-level temperature control method. Our simulation indicates that the temperature stability can reach 1.7 mK [5]. For the Laser Interferometer Space Antenna (LISA) project of the European Space Agency on gravitational wave detection, high-precision temperature measurements with a resolution at the micro-Kelvin level within the milli-Hertz frequency range are one of the primary requirements [6]. However, this is challenging due to the effect of factors such as resistance thermal noise and the inherent 1/f noise of electronic components.

In high-precision temperature measurements, platinum resistors and thermistors, whose resistance changes with temperature, are frequently used as temperature sensing elements. Many researchers have designed and manufactured temperature measuring systems for various applications using platinum resistors or thermistors. For example, Zhou et al. designed a multi-channel temperature measuring system with an error of less than 0.01 °C to meet the requirements for blackbody radiation surface temperature detection [7]. Based on platinum resistance and a bidirectional excitation constant current source, Hu et al. developed a temperature measurement system to meet the requirements of ultra-precision machining equipment [8]. The system demonstrated superior stability and resolution, better than 0.005 °C/10 days and 0.005 °C, respectively. Qin et al. designed a platinum resistance temperature measurement system utilizing a Wheatstone bridge and a reverse excitation voltage source to meet the requirements of submarine heat flow detection [9]. In situ seabed tests revealed a temperature fluctuation of 2.26 mK and a peak-to-peak resolution of 1.13 mK at 1.2~1.3 °C. Based on a Pt1000 platinum resistor, Zhang et al. developed a multi-channel low-power temperature measuring system that had a residual of 1 mK to meet the demands of thermal stress measurement [10]. However, the resolution of these temperature measuring devices is constrained by the noise emanating from components such as resistors, operational amplifiers, and power supplies, thus achieving only a milli-degree Kelvin level of precision. 

To achieve enhanced performance, extensive research has been undertaken on high-precision temperature measurement. The Fluke1560 stacked temperature inspection system can achieve a measuring resolution of 0.0001 °C when connecting platinum and thermistor probes [11]. Wudy et al. developed an negative temperature coefficient (NTC) thermistor temperature measuring system based on a voltage divider and DC technology, achieving a measurement frequency of 10 Hz and a resolution of 75 μK [12]. Zhang et al. designed a temperature measurement system characterized by a noise level of about 40 $\mu K/\sqrt{\mathrm{Hz}}$ in the frequency band of ≥0.5 mHz by driving a platinum resistor and a reference resistor with a sine wave signal and setting the central temperature point through a variable gain amplifier [13]. Guillet et al. developed a temperature reading system using an industrial platinum resistance temperature sensor. This system used an AC power supply and the lock-in amplifier method, achieving a system noise level of 25~30 $\mu K/\sqrt{\mathrm{Hz}}$ at 300 K and 1 Hz [14]. Sanjuán et al. designed an AC Wheatstone bridge temperature measurement system based on an NTC thermistor. The system achieved a noise-equivalent temperature of 1$0\;\mu K/\sqrt{\mathrm{Hz}}$ in the frequency range of 1 to 30 mHz at room temperature [15,16]. The system demonstrated satisfactory performance on the LISA Pathfinder mission, an advanced technology verification satellite for space gravitational wave detection [17,18]. Valentini et al. devised a temperature measurement and control system using platinum resistance. That system was employed in GOCE, a gravity field and ocean circulation detection satellite of the European Space Agency (ESA), achieving a temperature noise of 5$\;\mu K/\sqrt{\mathrm{Hz}}$ in the frequency band of 5 mHz~0.1 Hz [19,20]. This indicator is very high, but its technical details have not been reported yet.

Achieving temperature measurement resolution at the micro-Kelvin level within the milli-Hertz frequency range is exceedingly difficult due to resistance thermal noise and the inherent 1/f noise of electronic components. To solve this problem, this paper proposes a method based on an inductive voltage divider and an alternating current (AC) bridge. The features of this approach are as follows: (1) To overcome the influence of component’s 1/f noise, it uses an AC excitation signal to drive the temperature measuring bridge. Compared with the DC drive mode, it can prevent the noise of the DC voltage signal from being superimposed on the temperature change signal of the measurement output. (2) To reduce the influence of resistance thermal noise, it utilizes an LR AC bridge to realize temperature sensing and a current coupling amplification method. (3) It processes temperature change signals using a frequency domain algorithm based on the discrete Fourier transform, which can improve the accuracy of temperature measurement results. (4) It contains multiple sets of coil windings and reference resistors, which can ensure the balance degree of the AC bridge in a variety of temperature ranges, reduce the noise floor of the instrument, and improve the temperature measuring resolution. According to this method, a specific circuit was designed, manufactured, and tested. The results demonstrate that the noise level of the temperature measurement circuit is lower than $7\times 10^{-6}\;K/\sqrt{\mathrm{Hz}}$ in the range of 0.005~1 Hz, proving the effectiveness and feasibility of the proposed method.

## 2. Proposed Design Scheme

There are three methods for temperature measurement: voltage divider circuits [12,13], constant current sources plus series resistance [7,8,10], and bridge circuits [9,14,15,16]. The first two methods, which have low sensitivity and poor anti-interference capabilities, are frequently used in low-cost consumer electronics and industrial monitoring. Their measuring accuracy is affected by the stability of voltage source or current source. In contrast, the bridge circuit has the highest measuring accuracy. A typical bridge used for measuring resistance/temperature is the Wheatstone bridge, which consists of four resistors forming bridge arms and being connected in a closed loop. It applies excitation on one diagonal and measures the output at the other. The bridge circuit has many advantages: (1) high sensitivity, meaning it can detect small resistance changes; (2) differential output, which gives it a strong anti-interference ability; and (3) the ability to directly output voltage signals proportional to physical quantities, like temperature and pressure, making it suitable for dynamic measurements.

Thus, a bridge circuit is used in the paper. However, please note that we aim to achieve high-precision temperature measurements with resolution at the micro-Kelvin level in the frequency range of milli-Hertz. In this regard, controlling noise is the most challenging aspect. Because the fluctuations in the excitation source, the thermal noise of the resistor, the non-ideal characteristics of operational amplifier (e.g., thermal noise, 1/f noise, voltage noise, current noise, etc.), and other factors will adversely affect the measurement results, the question of how to suppress all kinds of noise has become the most critical issue. In light of this, a variety of measures have been taken in our proposed scheme. For example, in order to suppress 1/f noise, we use an AC signal as the excitation source of the bridge. In order to suppress the thermal noise of the resistors, we use as few resistive components as possible in the circuit design and propose replacing two voltage divider resistors with an inductive voltage divider in the bridge. Compared with division resistors, the inductive voltage divider has higher accuracy and no resistance aging issues because its division ratio depends on the winding turn ratio.

### 2.1. Temperature Measurement Circuit Based on Inductive Voltage Divider and AC Bridge

**Figure 1:**
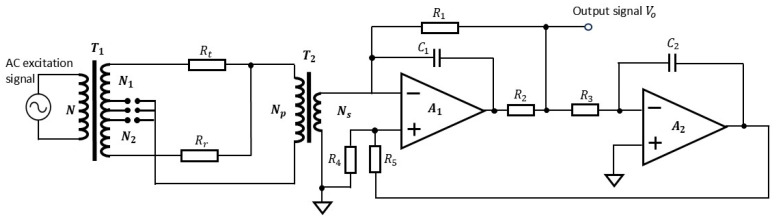
Schematic diagram of the proposed temperature measurement circuit.

Based on above considerations, we propose a temperature measurement circuit, as shown in Figure 1. We use two inductors (*N*_1_ and *N*_2_) formed by the two windings of inductive voltage divider *T*_1_, temperature sensing resistor *R_t_*, and reference resistor *R_r_* to form an AC bridge. The output of this bridge is coupled by a transformer and amplified by an operational amplifier to obtain output signal *V_o_*. At the same time, output voltage signal *V_o_* is fed back to the positive input of operational amplifier *A*_1_ after passing through an integration circuit and a resistance attenuation network to offset the influence of zero drift of the operational amplifier. In Figure 1, *T*_1_ is an isolated inductive voltage divider, with three windings wound around the same high-permeability magnetic core. An AC excitation signal is applied to the excitation winding with *N* turns; the other two windings have *N*_1_ and *N*_2_ turns respectively. When winding the inductive voltage divider, multiple taps can be set, as shown in Figure 1, and only one of the connections is selected according to actual needs during use. This will realize different voltage division ratios so as to make the bridge work in a good balanced state.

When the AC bridge is in a balanced state ($N_{1}R_{r}=N_{2}R_{t}$), the output of the bridge is 0. When the temperature change causes a variation in the resistance value of temperature sensing resistor *R_t_*, the AC bridge deviates from the balanced state, outputs a current signal related to the resistance variation of *R_t_*, i.e., the temperature change, and sends it to the subsequent signal detection circuit. As shown in Figure 1, the current signal is coupled by transformer *T*_2_ and converted to a voltage signal *V_o_* by a current/voltage conversion circuit composed of operational amplifier *A*_1_. Voltage signal *V_o_* is a quantity related to the excitation sine wave signal, circuit parameters, and the resistance value of the temperature sensing resistor, etc., and can be expressed as follows.

(1) $$V_{O}=\left(\frac{R_{r}}{R_{t}}-\frac{N_{2}}{N_{1}}\right)\frac{N_{1}}{R_{r}}\frac{U_{m}N_{p}}{NN_{s}}R_{1}$$

In Equation (1), *N* represents the number of turns of the excitation winding of inductive voltage divider *T*_1_, and *U_m_* is the AC excitation sine wave signal sent to the inductive voltage divider. *N*_1_ and *N*_2_ denote the turns of the other two windings of inductive voltage divider *T*_1_, respectively. *N_p_* and *N_s_* indicate the number of turns of the primary and secondary coils of transformer *T*_2_. *R_t_* refers to the resistance of the temperature sensing resistor, and *R_r_* refers to the resistance of the reference resistor. When the circuit board is manufactured, these parameters, such as *N*, *N*_1_, *N*_2_, *N_p_*, *N_s_* and *R_r_*, are all fixed and can be regarded as constants; only temperature sensing resistor *R_t_*, which is sensitive to the temperature variation of the environment to be measured, changes.

When a sine wave excitation signal is input to inductive voltage divider *T*_1_, the measurement circuit outputs temperature change signal *V_o_*, an analog sine wave signal with the same frequency as the sine wave excitation signal. This can reflect the change in resistance of the temperature sensing resistor, sense temperature variation, and achieve temperature measurement. Equation (1) shows the relationship between output voltage *V_o_* of the measurement circuit and temperature sensing resistor *R_t_*. From this equation, it can be inferred that output voltage *V_o_* of the circuit is directly proportional to excitation signal *U_m_*. By transforming Equation (1), we can obtain the expression that represents temperature sensing resistor *R_t_* using circuit output voltage *V_o_*:

(2) $$R_{t}=\frac{N_{1}}{\frac{N_{2}}{R_{r}}+\frac{NN_{s}}{N_{p}R_{1}}\frac{V_{o}}{U_{m}}}$$

Platinum resistors or thermistors are frequently used in high-precision temperature measurement applications. Considering that the target object we observe is the temperature of core components of a spacecraft whose variation range is narrow, we prefer to use a thermistor because it features high sensitivity, that is, it is more sensitive to temperature changes. This is conducive to the detection of small temperature changes in a narrow range. As is well known, the resistance of a thermistor is related to temperature, namely, the resistance of a thermistor will change when the temperature varies. The specific resistance–temperature relationship is as follows:

(3) $$R_{t}=R_{t25}e^{B\left(\frac{1}{T}-\frac{1}{T_{25}}\right)}$$

In this equation, *B* is the material constant of the thermistor, *T*_25_ represents 25 °C, and *R_t_*_25_ denotes the resistance value of the thermistor at 25 °C.

According to the relationship between the resistance value of temperature sensing resistor *R_t_* and temperature *T* to be measured (Equation (3)), as well as the relationship between temperature sensing resistor *R_t_* and output signal *V_o_* of the circuit (Equation (2)), we can write the expression of temperature *T* represented by *V_o_*, as shown in Equation (4), which indicates that the temperature value to be measured can be calculated from output voltage *V_o_* of the circuit.

(4) $$T=\frac{1}{\frac{1}{T_{25}}+\frac{1}{B}\ln\left(\frac{1}{R_{t25}}\cdot \frac{1}{\frac{N_{2}}{N_{1}R_{r}}+\frac{N_{s}}{N_{p}R_{1}}\cdot \frac{V_{o}}{U_{m}\cdot N_{1}}\cdot N}\right)}$$

Furthermore, by combining Equations (1) and (3), we can obtain the sensitivity of the circuit’s output *V_o_* to the temperature to be measured, as shown in Equation (5). It can be seen that the sensitivity of output *V_o_* of the circuit to temperature change is related to the AC excitation sine wave signal sent to inductive voltage divider *T*_1_, the turns ratio of the transformer *T*_2_, and the resistance value of resistor *R*_1_. Increasing the amplitude of the AC excitation sine wave signal or the resistance of resistor *R*_1_ is beneficial for improving measurement sensitivity.

(5) $$\frac{dV_{O}}{dT}=\frac{dV_{O}}{dR_{t}}\cdot \frac{dR_{t}}{dT}=\frac{R_{1}}{R_{t}}\frac{B}{T^{2}}\frac{N_{1}}{N}\frac{N_{p}}{N_{s}}U_{m}$$

### 2.2. Data Processing Algorithm Based on Discrete Fourier Transform

According to Equation (4), the temperature to be measured can be calculated from recorded output signal *V_o_* of the circuit. Because *V_o_* is a sine wave signal with the same frequency as AC excitation signal *U_m_*, we use a dedicated data acquisition device to simultaneously collect excitation signal *U_m_* on inductive voltage divider *T*_1_ and output signal *V_o_*. We then calculate the amplitude ratio of the two signals synchronously so as to eliminate the influence of excitation signal’s voltage fluctuation on the measurement results. After converting these two analog signals into two sets of digital sine wave voltage signal sequences using the data acquisition device, the amplitude of the two sets of digital sine wave signals is obtained through an amplitude measurement method based on discrete Fourier transform. The temperature value is then calculated according to the circuit parameters, and the ratio of the amplitude of digital output signal *V_o_* reflecting temperature changes to the amplitude of digital excitation signal *U_m_* (Equation (4)).

The collected digital output signal (a sine wave) is processed according to the following steps: ① Determine the frequency of the excitation signal (marked as *f_B_*) and the sampling rate of the data acquisition device (marked as *f_s_*); ② Segment the collected digital signal sequence at a time interval of t_1_ seconds, with a time window length of t_2_ seconds for each data section. In this way, adjacent time windows overlap by t_2_–t_1_ seconds; ③ Let the data in one time window of t_2_ seconds be a digital sine wave signal sequence *x*(*n*) with a length of $N_{a}$, and calculate its amplitude according to the discrete Fourier transform shown in Formula (6).

(6) $$X\left(k\right)=\frac{2}{N_{a}}\sum_{n=0}^{N_{a}-1}x\left(n\right)\left(\cos\frac{2\pi kn}{N_{a}}-i\sin\frac{2\pi kn}{N_{a}}\right)$$

We assume the frequency of sine wave signal is *f_B_*, and the sampling frequency of the data acquisition device is *f_s_*. When $k=N_{a}f_{B}/f_{s}$, *X*(*k*) is the complex value corresponding to the digital output sine wave signal with frequency *f_B_* (denoted as $X_{o}\left(k\right)$). For the collected digital excitation signal, we calculate its amplitude using the same amplitude measurement method, i.e., discrete Fourier transform (denoted as $X_{m}\left(k\right)$). Then, $V_{o}/U_{m}=\pm \left|X_{o}\left(k\right)\right|/\left|X_{m}\left(k\right)\right|,$ so we substitute the calculated $V_{o}/U_{m}$ into Equation (4) to get the temperature result. Here, if the phase angles of complex amplitude $X_{o}\left(k\right)$ and complex amplitude $X_{m}\left(k\right)$ are consistent, the sign on the right side of the equation is positive. If the phase angles of complex amplitude $X_{o}\left(k\right)$ and $X_{m}\left(k\right)$ differ by $180^{{}^{\circ}}$, the sign on the right side of the equation is negative.

For example, frequency *f_B_* of the excitation sine wave signal is 160 Hz, and sampling frequency *f_s_* of the data acquisition device is 500 Hz. For collected digital output signal $V_{o}$ and digital excitation signal *U_m_*, we truncate them at an interval of 0.2 s; the time window for each truncated data segment is 2 s. Therefore, the length of each truncated data segment is 1000, the adjacent data time windows overlap by 1.8 s, and $k=N_{a}f_{B}/f_{s}$ equals 320. By performing windowing and discrete Fourier transform on the truncated data segment, we get complex amplitude $X_{o}\left(320\right)$ of the digital output voltage signal with a frequency of 160 Hz as follows:

(7) $$X_{o}\left(320\right)=\frac{1}{500}\sum_{n=0}^{999}x_{o}\left(n\right)\left(\cos\frac{16\pi n}{25}-i\sin\frac{16\pi n}{25}\right)$$

Complex amplitude $X_{m}\left(320\right)$ of the digital excitation signal is calculated in the same way. Next, by calculating their modulus $\left|X_{o}\left(320\right)|/|X_{m}\left(320\right)\right|$, we can get the ratio of the amplitude of digital output signal *V_o_*, which reflects the temperature change to that of digital excitation signal *U_m_*, which we then substitute into Equation (4) to obtain the temperature value. Please note that by using the above method to continuously extract data and calculate temperature values, the resulting temperature sequence has a sampling rate of 5 samples per second (sps), i.e., a sampling interval of 0.2 s.

## 3. Results and Discussion

According to the above scheme, we design a specific temperature measurement circuit based on an inductive voltage divider and an AC bridge, draw the corresponding circuit schematic diagram and printed circuit layout, and select and purchase the components needed in the circuit, including the low-temperature-drift high-precision resistor used as a reference resistor for the AC bridge, the low-noise operational amplifier, etc. Then, we carry out the welding, debugging, and experiment work on the circuit board.

In this experiment, we choose a precision resistor produced by Vishay company as the reference resistor and select both nanocrystalline magnetic cores with high magnetic permeability and Teflon high-temperature wire to wind inductive voltage divider *T*_1_ and transformer *T*_2_. The inductive voltage divider for the AC bridge contains an excitation winding (60 turns) and three sets of coil windings (20 turns, 12 turns, and 12 turns respectively); the turns ratio of the primary and secondary coils of transformer *T*_2_ is 1:1. Next, the wound inductive voltage divider and the transformer are soldered onto the circuit board to form the AC bridge and the temperature measurement circuit.

We use a linear DC-regulated electric source (GPD-3303) to power the temperature measurement circuit board. The circuit board’s output (*V_o_*) which reflects temperature changes is sent to the computer in real-time by a dedicated data acquisition device for recording and display and saved in the form of a file. At the same time, we develop a computation program in the Matlab environment that implements data processing functions including amplitude measurement based on discrete Fourier transform (DFT), as described in Section 2.2, and temperature value conversion based on the resistance-temperature model, as described in Section 2.1. The input of the program is the recorded data file, and the output is the temperature value to be measured.

### 3.1. Noise Floor Level Test of the Temperature Measurement Circuit

In the laboratory, the ambient temperature is constantly changing. If there is no good thermostatic apparatus and a thermistor is directly used as the temperature sensing element, the measured results will always fluctuate with the ambient temperature, making it difficult to evaluate the indicators of the circuit board. Therefore, in this laboratory test, we replace temperature sensing resistor *R_t_* with a precision resistor to evaluate the noise floor of the designed temperature measurement circuit [15,16].

The room temperature in October is around 25 °C. The thermistor with a nominal value of 10 kΩ we plan to use has a resistance of nearly 3.3 kΩ at the gallium point. In order to simulate the working state of the thermistor at the gallium point, we choose a precision Vishay direct insertion resistor with a resistance of 3.3 kΩ as *R_t_* and select the two windings with 12 turns of induction transformer *T*_1_ as the two arms (*N*_1_ and *N*_2_) of the AC bridge. The reference resistor is composed of three Vishay direct insertion resistors connected in series/parallel, with a resistance of 3383 Ω.

**Figure 2:**
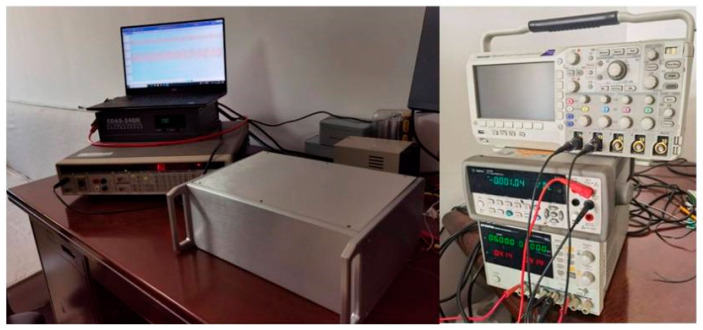
Experiment environment for noise floor level test at lab.

**Figure 3:**
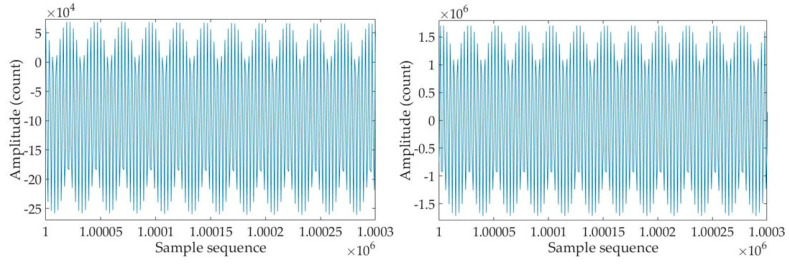
Output signal (**left**) and reference signal waveform (**right**) when sending a 10 Vpp 160 Hz sine wave excitation signal to the temperature measurement circuit.

Figure 2 shows the experimental environment for the noise floor level test at the lab. Firstly, we use a high-quality signal generator Model DS360 from Stanford Research System, Sunnyvale, CA, USA, to send a sine wave excitation signal with an amplitude of 10 Vpp and a frequency of 160 Hz to the temperature measurement circuit board. Specifically, it is sent to the excitation winding of inductive voltage divider *T*_1_, and the output of the circuit board is sent to the computer through a dedicated data acquisition device [21]. The output signal waveform and the reference signal waveform of the measurement circuit recorded on the computer are shown in Figure 3. As discussed in Section 2.1, the output signal waveform is proportional to the sine wave excitation signal, which means that the output is also a sine wave signal with a frequency of 160 Hz. The sampling frequency of the data acquisition device is set to 500 Hz. Due to the limits of the number of sampling points, it can be seen that the waveforms shown in Figure 3 are not very smooth, even though the shape of the sine waveform can still be observed. Furthermore, because the sampling frequency is higher than twice the frequency of the sine wave signal, the original sine wave signal can be completely presented from the recorded waveform data. The recovery of the subsequent sine wave signal and the calculation of the temperature value are not affected.

**Figure 4:**
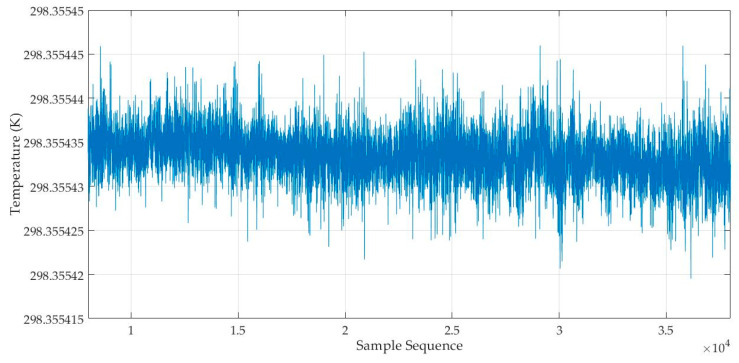
Calculated temperature value series when sending a 10 Vpp 160 Hz sine wave excitation signal by DS360.

According to Equation (4), the temperature value to be measured can be calculated from the recorded output waveform and reference waveform of the temperature measuring circuit. We substitute the specific values of circuit parameters *N*_1_, *N*_2_, *R_r_*, and *R*_1_ selected in this experiment into Equation (4), take the output signal waveform of the temperature measuring circuit as *V_o_* when a 10 Vpp 160 Hz sine wave excitation signal is input, and take the recorded waveform on the *N*_1_ winding as the reference waveform (i.e.,$\;U_{m}N_{1}/N$ in Equation (4)). Then, we calculate the temperature value to be measured using the Matlab (R2017b) program, developed according to the theory in Section 2. The result is shown in Figure 4. It can be seen that: ① The temperature value to be measured is about 25 °C, which is consistent with the laboratory environment temperature; ② The calculated temperature fluctuates within the range of 298.35542 K to 298.355445 K, with a very small fluctuation range of only 0.000025 K, which shows that the measurement result of this circuit is very good and the noise level of the circuit board is very low.

**Figure 5:**
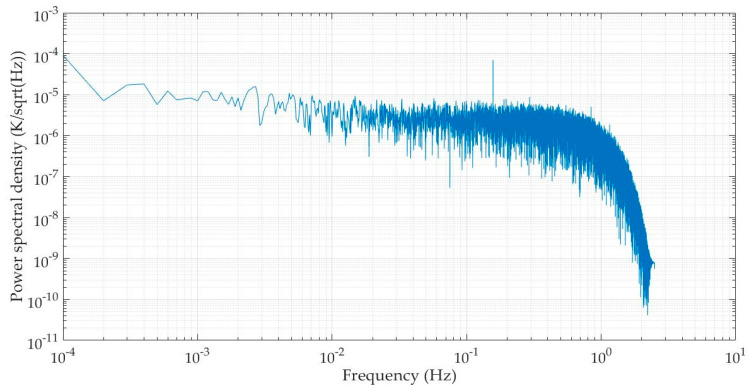
Power spectral density of temperature fluctuation data when sending an excitation signal by DS360.

To further investigate the noise level of the circuit board, we subtract the average from the obtained temperature value series to get the temperature fluctuation data during this period. Then, we generate its power spectral density diagram by applying the Pwelch function in Matlab, as shown in Figure 5, where the horizontal axis represents the frequency (in units of Hz) and the vertical axis represents the power spectral density of temperature fluctuation data (in units of K/sqrt(Hz). It can be seen that the noise floor of the temperature measuring circuit is better than $7\times 10^{-6}\;K/\mathrm{sqrt}\left(\mathrm{Hz}\right)$ in the range of 0.005~1 Hz. Within the same frequency band, Sanjuán et al. achieved a noise level of 10 $\mu K/\mathrm{sqrt}\left(\mathrm{Hz}\right)$ when using a fixed resistor instead of a temperature sensing resistor, a little larger than our result [16].

### 3.2. Temperature Test with Water Three-Phase Point Bottle

Considering the narrow temperature variation range of the observation object, we use a thermistor as the temperature sensing element, because it has high sensitivity, that is, it is more sensitive to temperature change, which is conducive to the detection of small temperature variations within a narrow range. The resistance of the thermistor we plan to use in our experiment is 11,786.7206 ohms at the water three-phase point. According to this thermistor value, we select the two windings of 32 and 10 turns of the inductive voltage divider as the two arms of the AC bridge and select several Vishay direct-inserted resistors, which are connected in series/parallel, to form the reference resistor with a resistance of 4420 ohms.

**Figure 6:**
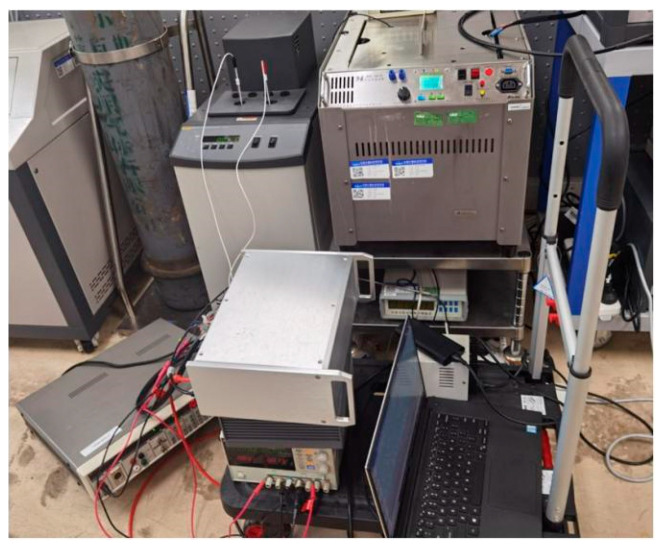
Test environment with water three-phase point bottle at the National Institute of Metrology, China.

This temperature test experiment with the water three-phase point bottle is carried out at National Institute of Metrology, Beijing, China. The temperature sensing element–thermistor is put in a slender glass tube with an oily substance at the bottom, and the glass tube is placed into the water three-phase point bottle (Figure 6). A sine wave excitation signal with an amplitude of 10 Vpp and a frequency of 160 Hz is sent to the excitation winding of the inductive voltage divider. The output of the circuit board and the reference signal are sent to the computer for real-time display, recording, and analysis via a dedicated data acquisition device.

**Figure 7:**
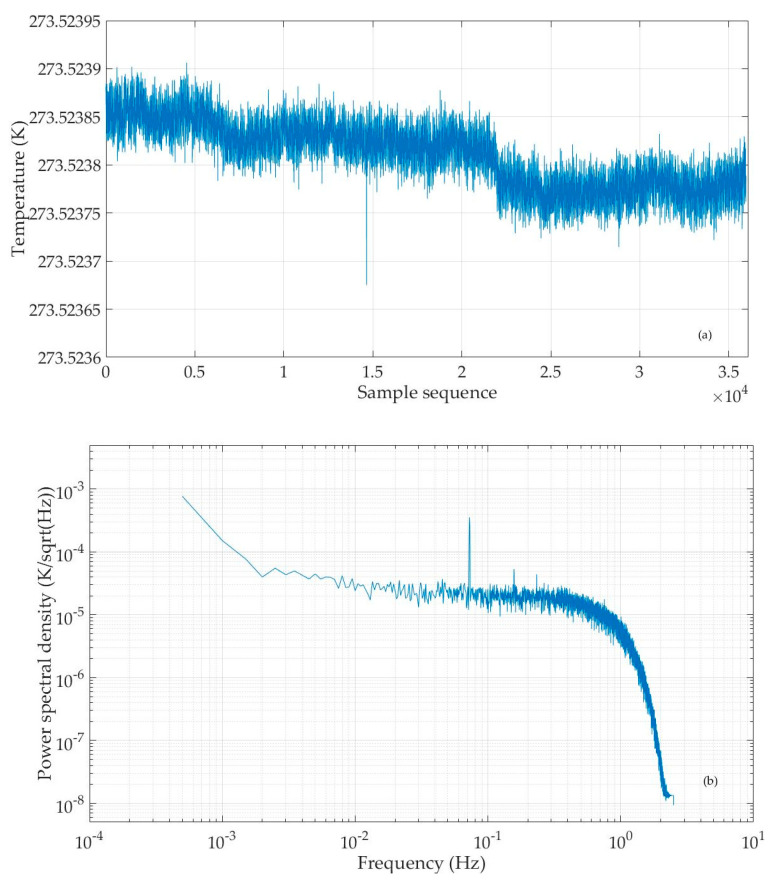
Calculated temperature value series (**a**), and power spectral density of temperature fluctuation data (**b**) when sending a 10 Vpp 160 Hz sine wave excitation signal to the circuit, with the thermistor in water in a three-phase point bottle.

As in the previous experiment, we use the developed computation software to calculate the temperature value to be measured from the recorded circuit’s output and reference signal. The result is shown in Figure 7a. The temperature value fluctuates between 273.5237 K and 273.5239 K. Next, we subtract the average from the temperature value series to obtain the temperature fluctuation data and then use the Pwelch function to compute the power spectral density of temperature fluctuations. In Figure 7b, the horizontal axis represents frequency (in units of Hz) and the vertical axis represents the power spectral density of temperature fluctuation data (in units of K/sqrt(Hz)). From this result, it can be seen that when the temperature sensing element is a thermistor, the noise level of the temperature measurement circuit is 20~30 $\mu K/\mathrm{sqrt}\left(\mathrm{Hz}\right)$ within the range of 0.01~1 Hz, i.e., better than the research results of predecessors [7,8,9,10,11,12,13,14]. It should be noted that in this figure, a small frequency component resembling a weak signal can be seen. This may be due to the lack of electromagnetic shielding measures in the experiment, resulting in the circuit board being affected by the power frequency and electromagnetic interference.

### 3.3. Discussion

As shown in Figure 1, inductors (*N*_1_ and *N*_2_) formed by two windings of inductive voltage divider *T*_1_, temperature sensing resistor *R_t_*, and reference resistor *R_r_* are used to compose an AC temperature measuring bridge. The output of this bridge is amplified by a transformer coupling and an operational amplifier to get output signal *V_o_*, which reflects temperature change. For this temperature measurement circuit, the main noise contribution comes from the reference resistor, the temperature sensing resistor, and the operational amplifier. Therefore, in this experiment, attention should be paid to the selection of these components.

**Figure 8:**
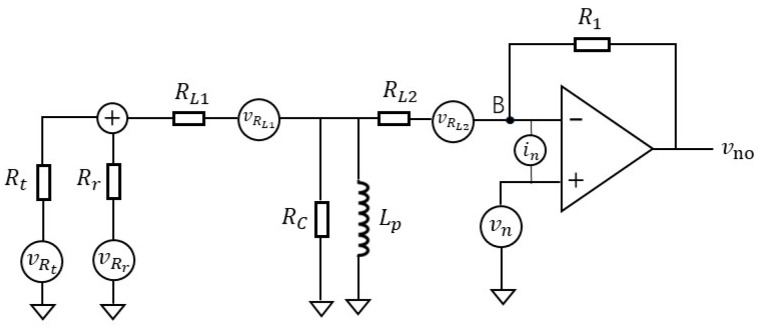
Noise model of the temperature measurement circuit.

Figure 8 illustrates the noise model of the temperature measurement circuit. In this model, $v_{Rt}$ and $v_{Rr}$ represent the thermal noise of the temperature sensing resistor and the reference resistor respectively; $v_{n}$ is the voltage noise, and $i_{n}$ refers to the current noise of the operational amplifier. The other part shows the equivalent model of transformer *T*_2_ at low frequency [22], where *R_L_*_1_ and *R_L_*_2_ are the internal resistances of the primary and secondary coils of the transformer, respectively, *L_p_* is the inductance of the primary coil of the transformer, and *R_C_* is the equivalent resistance of the transformer core loss, which is very large and can be regarded as an open circuit. $v_{RL1}$ and $v_{RL2}$ denote the thermal noise of coil internal resistances *R_L_*_1_ and *R_L_*_2_.

In the experiment of Section 3.1, the resistance of temperature sensing resistor *R_t_* is 3300 ohms, and that of reference resistor *R_r_* is 3383 ohms. The resistances of both *R_L_*_1_ and *R_L_*_2_ are 1.12 ohms, which is about one in three thousand of the temperature sensing resistance and reference resistance. Compared with the temperature sensing resistor and reference resistor, the resistance of the coil (*R_L_*_1_ and *R_L_*_2_) is very small, and its influence can be ignored. Therefore, in order to facilitate the calculation and reduce the complexity, we neglect resistors *R_L_*_1_ and *R_L_*_2_, as well as their thermal noise, in the subsequent analysis.

Next, we derive the magnitude of noise voltage at the output of the circuit generated by the temperature sensing resistor noise, the reference resistor noise, the operational amplifier’s voltage noise, and the current noise. Since the current circuits of the temperature sensing resistor and the reference resistor are independent, their noise should be calculated separately. We first consider the transfer function of thermal noise of the temperature sensing resistor (marked as $v_{Rt}$). Assuming that the output noise value caused by the thermal noise of this resistor is $v_{1}$, Equation (8) can be obtained at node B of Figure 8 according to Kirchhoff’s current law. Here, *A*_0_ is the open-loop amplification factor of the operational amplifier; then, $A_{0}v_{-}=v_{1}$ for the operational amplifier. By replacing $v_{-}$ by $v_{1}$ in Equation (8), we can get the mathematical expression as Equation (9), which denotes the relation between $v_{Rt}$ and $v_{1}$.

(8) $$\frac{v_{Rt}-v_{-}}{R_{t}}-\frac{v_{-}}{R_{r}}-\frac{v_{-}}{sL_{p}}+\frac{v_{1}-v_{-}}{R_{1}}=0$$

(9) $$\frac{v_{Rt}}{R_{t}}=\frac{v_{1}}{A_{0}}\left(\frac{1}{R_{t}}+\frac{1}{R_{r}}+\frac{1}{sL}+\frac{1}{R_{1}}\right)-\frac{v_{1}}{R_{1}}$$

In this experiment, the resistance of temperature sensing resistor *R_t_* is 3300 ohms, and that of reference resistor *R_r_* is 3383 ohms. The inductance of *L_p_* is 2 H, and the resistance of *R_1_* is 20,000 ohms. *A*_0_, the open-loop amplification factor of the operational amplifier, is very large, i.e., usually between 10^6^ and 10^7^. This makes the first term on the right side of Equation (9) much smaller than the second term, so we can ignore the first term and get the expression of output noise value $v_{1}$ using Equation (10).

(10) $$v_{1}\approx -\frac{R_{1}}{R_{t}}v_{Rt}$$

Assuming that the output noise value caused by the thermal noise of reference resistor is $v_{2}$, the mathematical expression of $v_{2}$ can be obtained in the same way, as shown in Equation (11).

(11) $$v_{2}\approx -\frac{R_{1}}{R_{r}}v_{Rr}$$

The thermal noise of the 3300 ohms temperature sensing resistor is 7.4 nV/sqrt(Hz), and that of the 3383 ohms reference resistor is 7.5 nV/sqrt(Hz). Substituting them into Equations (10) and (11), we can see that thermal noise output values $v_{1}$ and $v_{2}$ are 44.8 nV/sqrt(Hz) and 44.3 nV/sqrt(Hz), respectively.

Suppose the output noise value caused by the voltage noise of the operational amplifier is $v_{3}$. According to Kirchhoff’s current law and the virtual short property of the input terminal of the operational amplifier, the output value of the voltage noise of the operational amplifier can be expressed as follows.

(12) $$v_{3}=\left(1+R_{1}\left(\frac{1}{R_{t}}+\frac{1}{R_{r}}+\frac{1}{sL}\right)\right)\cdot v_{n}$$

Suppose the output noise value caused by the current noise of operational amplifier is $v_{4}$, then its mathematical expression is:

(13) $$v_{4}=i_{n}{\left(\frac{1}{R_{t}}+\frac{1}{R_{r}}+\frac{1}{sL}+\frac{1}{R_{1}}\right)}^{-1}$$

We select the Analog Devices Corporation’s ADA4084-1 as the operational amplifier for this experiment. According to the datasheet, its voltage noise density ($v_{n}$) is 4 nV/sqrt (Hz) @ 160 Hz, and its current noise density ($i_{n})$ is 0.55 pA/sqrt(Hz). By substituting these values into the above two equations, we obtain that the noise output value $v_{3}$ caused by voltage noise of the operational amplifier is 65.4 nV/sqrt (Hz), and noise output value $v_{4}$ caused by current noise of the operational amplifier is 0.7 nV/sqrt (Hz). By superimposing the power of noise output values $v_{1}$, $v_{2}$, $v_{3}$, and $v_{4}$, it can be determined that the total output noise of the circuit is 90.8 nV/sqrt(Hz) and the equivalent temperature noise floor is 0.5 $\mu K/\mathrm{sqrt}\left(\mathrm{Hz}\right).$ The measured noise floor of our circuit board, shown in Figure 5, is below 7 $\mu K/\mathrm{sqrt}\left(\mathrm{Hz}\right)$ in the frequency band of 0.005~1 Hz, i.e., higher than the result calculated by the noise model. This is reasonable, because in practical experiment, there are many other factors that may affect the measurement result.

**Figure 9:**
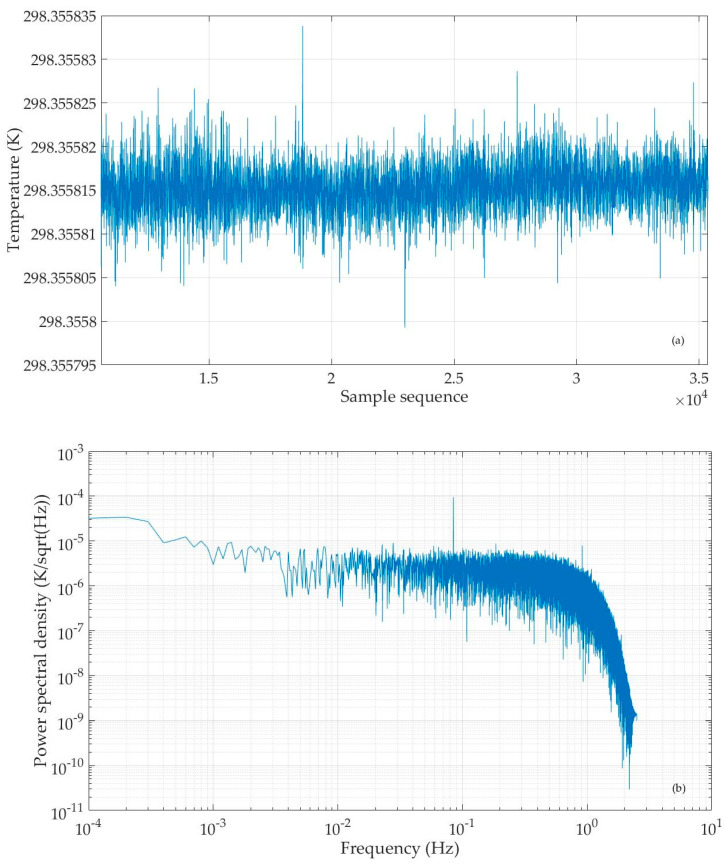
Temperature value series (**a**) and power spectral density of temperature fluctuation data (**b**) when sine wave excitation signal is generated by CPU and DAC chips.

In the above experiment, we use a DS360 low-distortion signal generator to provide a sine wave excitation signal to the temperature measurement circuit board. However, in an actual application, it is impossible to configure a signal generator for each temperature measuring device because of the high cost. Sine waves are often generated by the LC/RC oscillator circuit or by a CPU chip plus DAC. Here, we apply a CPU chip (stm32L431) and a 16-bit DAC chip to generate a sine wave and send it as an excitation signal to the circuit board for testing. As in Section 3.1, we calculate the temperature value to be measured and the power spectral density of the temperature fluctuation data by using the same method and program. The results are shown in Figure 9. It can be seen that the noise floor level of the circuit board measured by using the sine wave generated by the CPU and DAC chips as the excitation signal is almost the same as that measured using the sine wave generated by the DS360 signal generator. This shows that the sine wave generated by the CPU and DAC chips is of good quality and can be used as the excitation signal in practical application to provide a power supply for the AC bridge of the temperature measuring circuit.

Attention should be paid to the balance degree of the AC bridge, because this influences the measurement results; the better the balance of the bridge, the better the temperature measurement effect. We have fully considered the importance of balance degree in our experiments by setting multiple sets of coil windings in *T*_1_ and multiple reference resistors. In summary, the results and analysis show that our designed temperature measurement circuit performs well and has great application potential. We will next integrate temperature detection, A/D conversion, and an embedded system to form a practical, low-noise and high-precision temperature measuring instrument.

## 4. Conclusions

We propose a temperature measurement technique based on an inductive voltage divider and an AC bridge to satisfy the space gravitational wave detection requirement for temperature detection. We accomplish this by taking the following two steps to create a low-noise, high-precision temperature measurement system: (1) The inductors formed by two coil windings of an inductive voltage divider, a temperature sensing resistor, and a reference resistor are utilized to compose the AC bridge, and a transformer is applied to complete current coupling in the signal detection circuit. In order to overcome the effects of resistance thermal noise and intrinsic low-frequency (1/f) noise within the components, we minimize the use of resistors as much as possible and employ an AC excitation signal to drive the temperature measurement bridge. This contributes the most to performance improvement. (2) A frequency domain algorithm based on Fourier transform is used to process the recorded temperature change data to improve the accuracy of the temperature measurement results. We create a specific circuit board and carry out tests. The results show that the noise floor of the temperature measurement circuit is better than $7\;\mu K/\sqrt{\mathrm{Hz}}$ in the range of 0.005–1 Hz. When using a thermistor as a temperature sensing component in practice, its noise level is $20\sim 30\;\mu K/\sqrt{\mathrm{Hz}}$ within the range of 0.01~1 Hz. These results verify the feasibility and effectiveness of the proposed design scheme. It is expected that this method will have a wide range of potential applications in fields like aerospace [23], precision measurement [24,25], semiconductor manufacturing [26,27], ocean exploration [28,29], and geophysics [30,31], all of which have high demands regarding noise level and temperature measurement resolution.

## Data Availability

Data are contained within the article.
